# The Subjective Sensation of Synchrony: An Experimental Study

**DOI:** 10.1371/journal.pone.0147008

**Published:** 2016-02-12

**Authors:** Joan Llobera, Caecilia Charbonnier, Sylvain Chagué, Delphine Preissmann, Jean-Philippe Antonietti, François Ansermet, Pierre J. Magistretti

**Affiliations:** 1 Immersive Interaction Group, EPFL, Lausanne, Switzerland; 2 Medical Research Department, Artanim Foundation, Geneva, Switzerland; 3 Institute of Psychology, University of Lausanne, Lausanne, Switzerland; 4 Department of Ecology, University of Lausanne, Lausanne, Switzerland; 5 Department of Child and Adolescent Psychiatry, University Hospital of Geneva, Geneva, Switzerland; 6 Department of Psychiatry, University of Geneva, Geneva, Switzerland; 7 KAUST, Thuwal, Makkah Province, Saudi Arabia; 8 Brain Mind Institute, EPFL, Lausanne, Switzerland; 9 Agalma Foundation,Geneva, Switzerland; University of Reading, UNITED KINGDOM

## Abstract

People performing actions together have a natural tendency to synchronize their behavior. Consistently, people doing a task together build internal representations not only of their actions and goals, but also of the other people performing the task. However, little is known about which are the behavioral mechanisms and the psychological factors affecting the subjective sensation of synchrony, or “connecting” with someone else. In this work, we sought to find which factors induce the subjective sensation of synchrony, combining motion capture data and psychological measures. Our results show that the subjective sensation of synchrony is affected by performance quality together with task category, and time. Psychological factors such as empathy and negative subjective affects also correlate with the subjective sensation of synchrony. However, when people estimate synchrony as seen from a third person perspective, their psychological factors do not affect the accuracy of the estimation. We suggest that to feel this sensation it is necessary to, first, have a good joint performance and, second, to assume the existence of an attention monitoring mechanism that reports that the attention of both participants (self and other) is focused on the task.

## Introduction

People performing actions together have a natural tendency to synchronize their behavior. This occurs spontaneously, even when not requested to do so, and in a variety of contexts, either in tasks involving a shared goal [1, 2], or in more freeform tasks [3], even without being aware of it.

In addition, behavioral synchronization is also accepted as a mechanism supporting social interaction [4]. Dyads with strong interpersonal coordination are perceived as having more rapport [5], people with more pro-social orientation will synchronize more easily [6], altruistic behavior in kids can be promoted with sessions of interpersonal coordinated behavior [7] and people with deficits in social behavior have shown lower spontaneous interpersonal synchronization [8]. Consistently, people doing a task together build internal representations not only of their actions and goals, but also of the other people performing the task [2]. In addition, when the task involves an explicit requirement to synchronize, the stability of the synchronization is modulated by attention [9].

At a physiological level, spontaneous autonomic responses reflect joint performance [3]. It has also been shown that interbrain synchronization is present when two people are involved in a joint sensorial or sensorimotor task (see, for example, Dumas, Nadel [10]). However it is unclear whether this synchronization is merely a by-product of having two people doing the same task or perceiving the same stimuli, or whether the synchronization really reflects a joint shared representation of the actions of both participants [11].

From a behavioral perspective, it has also been shown that people will sometimes perform better when relying on these coordination mechanisms rather than a hierarchical relation based on leader and imitator [12]. Respiration synchronization has also been found in choir singing [13], or, between bystanders and actors involved in collective rituals [14]. This suggests that tasks inducing the subjective sensation of ‘mutual connection’ might involve a stronger level of coordination and could play a role, for example, in joint or collective creativity [15].

However, little is known about which are the factors inducing the subjective sensation of synchrony, or ‘connecting’ with someone else. One option would be that the subjective sensation of synchrony is a mere consequence of interpersonal behavioral synchrony. However, this is unlikely since external factors can also affect this feeling. For example, in a study on spontaneous interpersonal synchronization, music has been shown to act as a social glue: performing the same task with music resulted in stronger feelings of synchrony, despite not inducing stronger behavioral synchronization [16]. It is also possible that interpersonal factors contribute to the subjective sensation of synchrony. We propose as possible interpersonal factors affecting such sensation the empathic abilities of the participants involved, or their affective state. Alternatively, it is also possible that the subjective sensation of synchrony, when no music or other external factor can be used as a reference, requires a joint task and a shared goal to arise.

Here we address the question of what factors are involved in inducing a subjective feeling of synchrony when two or more people are performing a task together. For this purpose, we devised a task reliably inducing a sensation of synchrony. We adapted a mirror imitation task [12, 15] to involve full body interaction. People were simply asked to perform a joint task with their arms, while experiencing the actions from their own natural first-person perspective, and report when they felt they were performing in synchrony with the other. To assess whether the subjective feeling of synchrony was related to spontaneous synchrony, we also introduced an implicit synchronization task, prior to the main task. In addition, to assess the reliability of this subjective sensation, and whether it was equivalent to the estimation of behavioral synchrony, asked people, on a later session to report when they thought this sensation occurred in motion videos, as seen from a third-person perspective.

## Methods

### Participants

Twenty healthy right-handed subjects (10 males, 10 females, mean ± standard deviation age: 33.8 ± 10.7 years) were randomly assigned an unknown partner to form a male-female dyad for the study. All participants provided written informed consent after receiving a detailed explanation of the experimental procedures, and they received a nominal fee for their participation. They were recruited through advertisements posted in local universities as well as online. Participants were excluded if they had a history of neurological or mental disorder such as seizure, stroke, mood disorder or depression. The Institutional Review Board of the University Hospitals of Geneva approved all experimental procedures for this study.

### Materials

**Fig 1:**
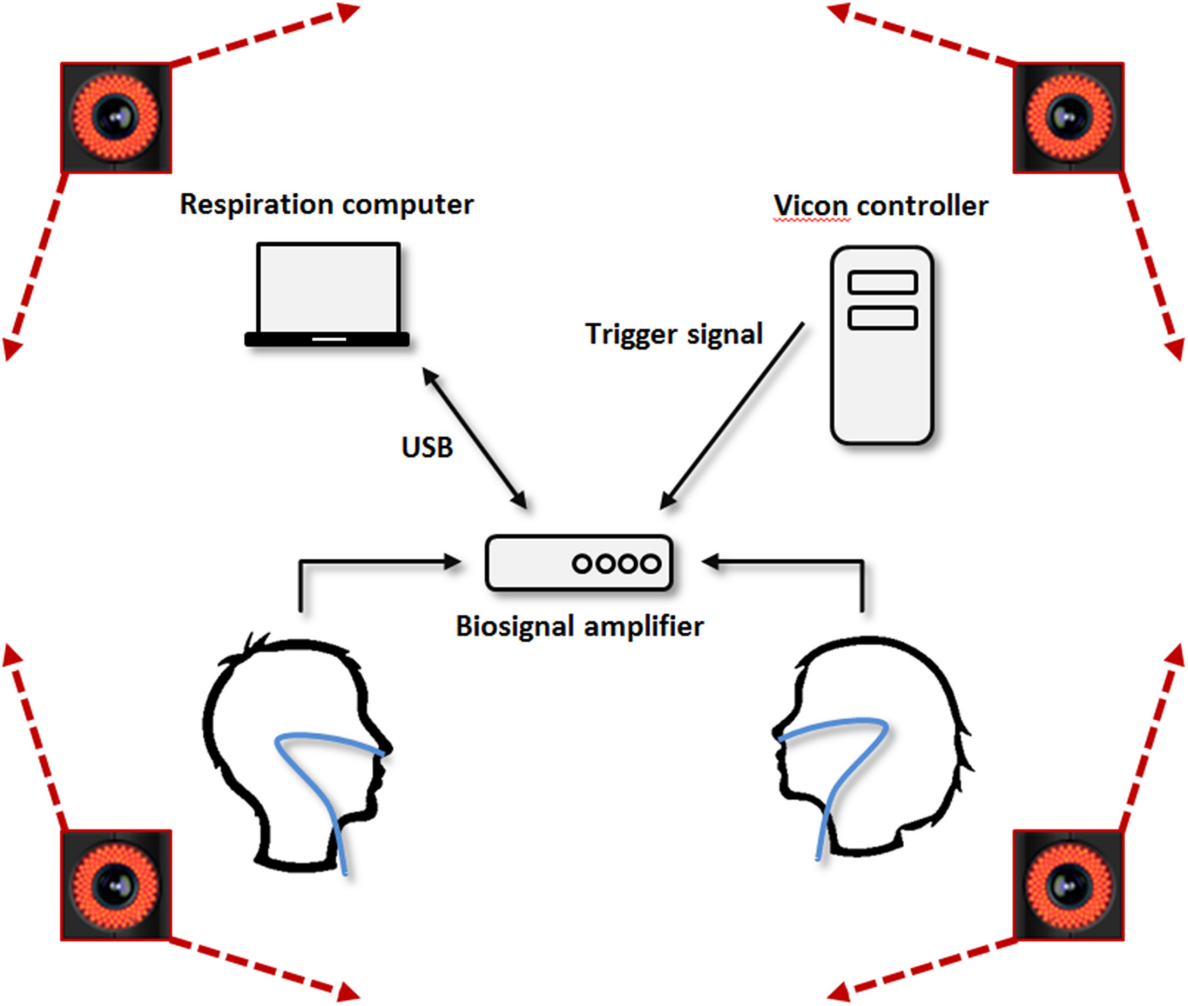
Hardware setup: the respiration data of the two participants was passed through a biosignal amplifier to the respiration computer. The Vicon controller received body motion data from the participants. The two systems were synchronized using a digital signal sent from the Vicon controller to the respiration computer through the digital input channel of the biosignal amplifier.

**Fig 2:**
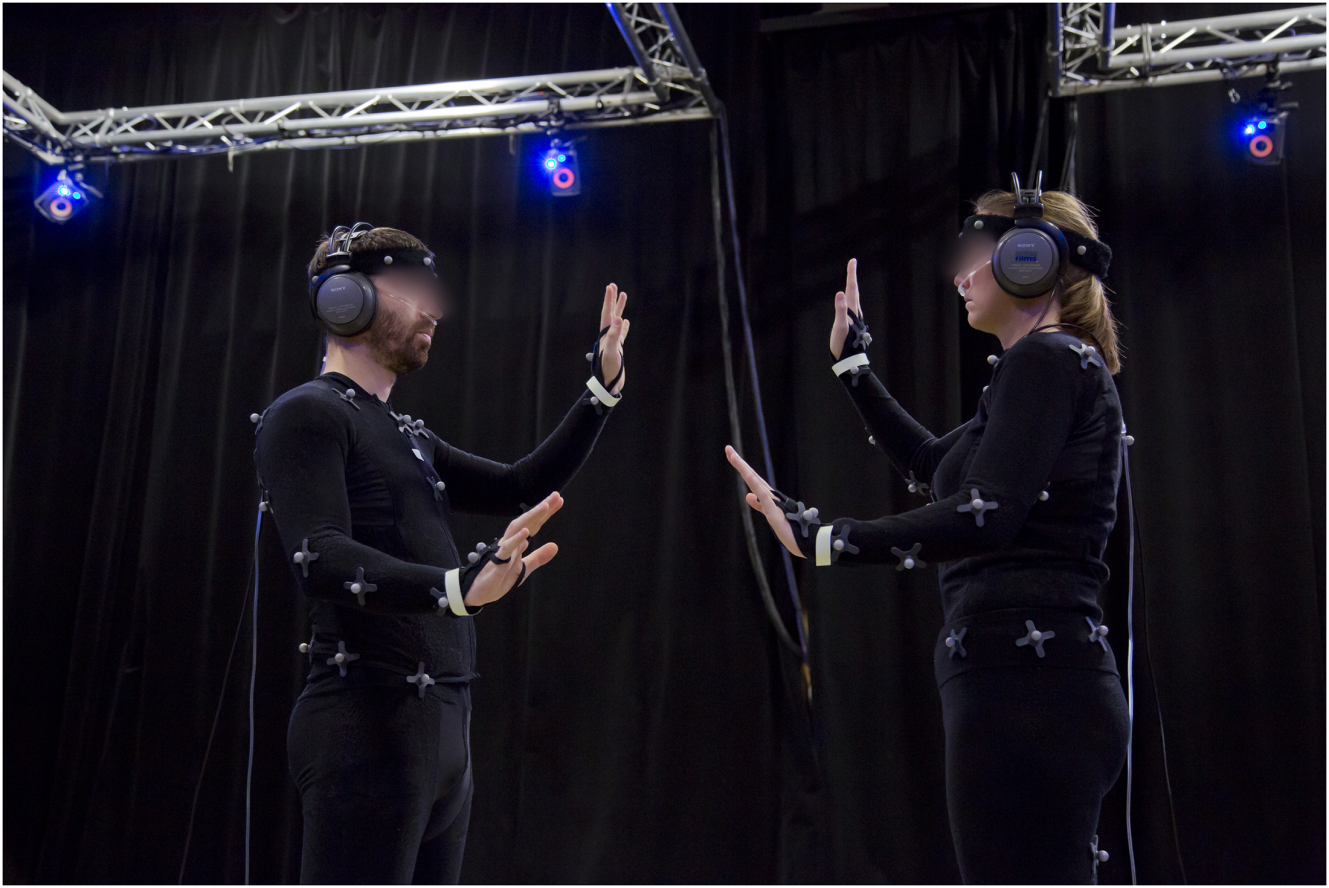
The experimental setup during the mirror game. Couples of participants were asked to play the mirror game by performing movements together (*joint* condition) or without seeing each other (*blind* condition). They would individually report the instant when the sensation of synchrony appeared. It should be noted that for illustration reasons the light is on but the tests were performed in the dark. In the real conditions, participants could not see each other, only the phosphorescent tape placed on the chest and wrists were visible.

A schematic representation of the hardware setup is shown Fig 1. Fig 2 illustrates the experimental setup used during the experiment.

A Vicon MXT40S motion capture system (Vicon, Oxford Metrics, UK) consisting of ten infrared cameras tracked the movements of reflective markers (Ø 14 mm) at a sampling rate of 120 Hz. Data were captured within a 26.9 m^3^ measurement volume (4 × 2.8 × 2.4 m). The participants were equipped with a Velcro^™^ motion capture suit and with 53 markers placed on each body’s joint to track their movements during the experiment. The standard Vicon Blade marker set (Vicon, Oxford Metrics, UK) was used. This marker set allows gathering motion data for the following joints: neck, trunk, pelvis, shoulders, elbows, wrists, knees and ankles.

To prevent biases due to facial expressions or other visual cues, the motion experiment was executed in the dark. To act as fixation point, one phosphorescent tape (Wellys, reflective safety tape) of 2 cm width and 4 cm length was fixed on the torso. In addition, to monitor each other’s behavior, two phosphorescent tapes of 2 cm width were placed around the wrists of each participant using Velcro^™^ straps. Participants were also isolated from ambient noise and verbal cues by wearing headphones with continuous white noise. The sound level was adjusted for each participant to their maximum level of tolerance, and it was verified that they were not able to hear the voice of their partner. More details about the experimental procedures are given in the next section.

Since it is known that respiration synchrony relates to spontaneous behavioral synchrony, we also measured respiration for each participant. Respiration was collected at 256 samples per second using a thermistor-based SleepSense Flow sensor placed beneath the nose. The sensor was connected to a biosignal amplifier (g.USBamp, g.tec, Schiedlberg, Austria) which had a 30.0 Hz low-pass filter and a 0.1 Hz high-pass filter, as well as a 50 Hz notch filter to suppress the power line interference. The respiration data was sent via USB to a dedicated laptop, and a Simulink^®^ (The MathWorks Inc.) program was implemented for display and storage.

The motion capture data acquisition was controlled by a separate computer (Vicon controller). To synchronize the motion capture data and the respiration data, the Vicon controller and the laptop capturing the respiration data were connected together through a trigger cable. To send trigger signals from the Vicon controller to the laptop, we developed a custom code integrated within the Vicon Blade software. This allowed us to start/stop the motion capture from the Blade graphics user interface (GUI) and send automatically trigger signals to mark specific instants in the movement and respiration data logs. FThe experimenter had, therefore, 6 buttons: 2 buttons to start and stop the motion recording and four additional buttons to record the state of each of the two participants: one button for "sensation of synchrony appears" and one button for "sensation of synchrony disappears". This allowed to translate the oral response of each participant to an event in a log file. Keyboard shortcuts were also implemented to reduce as much as possible the latency in the reporting.

To assess subjective factors, we selected a widely used empathy questionnaire [17] to extract an empathy score for each participant. The twenty questions of the Positive and Negative Affect Schedule (PANAS) questionnaire [18, 19] were used as a state measure of positive and negative affect.

### Design and procedure

The experiment was divided in two parts: a motion experiment and a video test session conducted two months later. Upon arrival the two participants completed the empathy questionnaire and the PANAS questionnaire to measure the positive and negative affective state before the movement tasks.

Then, participants were equipped with the motion capture suit (see Fig 2) and instructed to walk in a circle indicated on the floor, starting at opposite sides of the circle. Participants were told to walk in the same direction at a self-selected speed. They were also told that the task was necessary for calibrating the motion capture system, even if the true objective was to measure their ability to spontaneously synchronize during walking, through an implicit measure. The experimenters counted seven laps before stopping the task.

Next, the participants were equipped with the respiration sensor and the headphones. They were placed standing opposite one another with a face-to-face distance of 80 cm to ensure that no physical contact could be made (see Fig 1). If one of the participants was smaller than the other, their height was adjusted using a polystyrene block, so that their shoulders met at the same height. They performed two successive motion sessions in two different conditions, *blind* and *joint*, each consisting of 6 motion tasks.

For the *blind* condition, the participants executed the tasks with a sleep mask placed on their eyes in order to perform the task without being distracted by the other participant’s movements. The aim of this session was to gather baseline motion data on how they performed the tasks individually, without feedback from the other participant.

For the *joint* condition, the participants were asked to execute the tasks mirroring each other and in a continuous and synchronized manner—the goal being to have both hands at the same spatial positions as the ones of their partner. In order for the participant to not be influenced by his partners’ facial expression, appearance, or other visual factors, the overhead lights were turned off and the tasks were carried out in the dark. Visual cues were provided using phosphorescent tape attached around the wrists of each participant. To prevent biases due to head motion or distraction, participants were instructed to look at the other participant’s phosphorescent tape fixed on the torso during the whole duration of the task. In addition, after each 1 minute period of task performing the lights were turned on again to prevent adaptation to dark.

While performing the task, participants were also instructed to say aloud when they felt the sensation of being in synchrony with their partner, and when this sensation disappeared. For simplicity and clarity, the participants said their ‘first name’ to report the appearance of the sensation and ‘NOT first name’ to report its disappearance. One of the experimenters would then use a keyboard to log the subjective report synchronized with the physiological and motion data.

Participants were unable to distinguish the other’s lip motion during the announcements and the voice or any other sound was covered by the white noise. The task was validated only if both participants reported the sensation of synchrony during a common phase of at least 10 seconds; otherwise the task was repeated before continuing to the next (maximum of 4 attempts).

**Fig 3:**
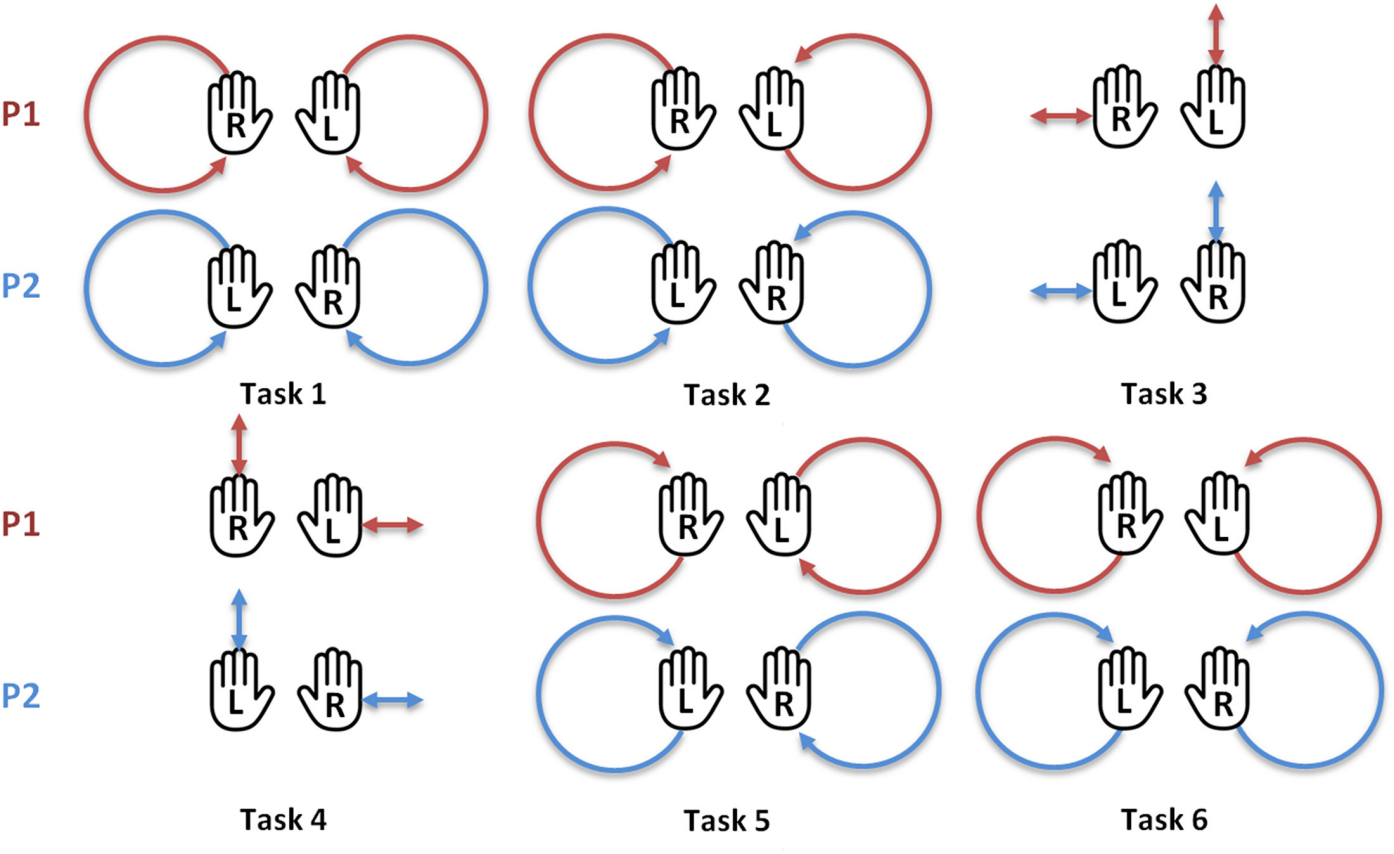
The six motor tasks performed by the two participants. During the *blind* and *joint* motion sessions each task instructed to the first participant (P1) was executed in mirror by the second participant (P2). R = right hand, L = left hand.

Each session included six different motor tasks using the upper body only (Fig 3), as follows:

- Task 1: Participant 1 (P1) and participant 2 (P2) move the right hand in a clockwise circle and the left hand in a counterclockwise circle;
- Task 2: P1 moves both hands in clockwise circles, P2 moves both hands in counterclockwise circles;
- Task 3: P1 moves the right hand horizontally and the left hand vertically, P2 moves the right hand vertically and the left hand horizontally;
- Task 4: same as the task 3, but the participants’ moves are reversed;
- Task 5: same as the task 2, but the participants’ moves are reversed;
- Task 6: same as the task 1, but the participants’ moves are reversed;

The participants were instructed to start the tasks with both hands clenched in front of them. Each task lasted 1 minute. The minimum frequency imposed to the subjects was around 0.3Hz, but they could choose their own frequency to perform the task. To prevent an order effect, both the order in which the conditions (*blind*, *joint* vs. *joint*, *blind*) and the order of the motor tasks (tasks 1–6 vs. tasks 6–1) were counterbalanced between couples.

The motion capture session was split in two blocks (*blind*, *joint* or, *joint*, *blind*), separated by a 10 minutes break. In each block, participants would do all 6 tasks, either in forward (from 1 to 6) or reverse order (6 to 1).

During this break as well as after the second motion session, participants were asked again to complete the PANAS questionnaire to measure if their mood changed during the experiment. Therefore, PANAS questionnaires were used three times to assess the evolution of emotional activations through the experiment.

Two months later, participants were recalled individually for the second part of the experiment. They were invited to watch 42 videos of the motion experiment in a random order—the 6 videos of their own performances during the *joint* condition session and 36 videos of other dyads (6 dyads × 6 tasks) randomly chosen. Three dyads were hence excluded from the viewing in order to limit the time required to complete the test and to avoid false responses due to fatigue. Each video lasted 1 minute and contained a point light display [20, 21] consisting of 21 moving dots against a black background and showing the dyad during the entire motor task from an angle of approximatively 45° from the back of the first member of the dyad (S1 Video). The kinematic information was thus preserved but without the features and visual information. For each video, participants were asked to indicate: Q1) the starting time (time code) when they thought that the two people were in synchrony; Q2) if they were present in the video; and Q3) to which extent they were confident in their response to question 2 (0: not sure at all, 10: completely sure). This last question was conceived as a way to obtain a more nuanced input to their response in Q2. The test was performed on a standard laptop using an external 22 inch LCD monitor. The videos were played in full-screen mode and were presented in a playlist using a common video player (VLC, VideoLAN). Participants were free to replay the videos, go back and forward, and had to write their responses on paper.

### Data processing

The three-dimensional motion data was post-processed in Vicon Blade software (Vicon, Oxford Metrics, UK) and the marker trajectories were exported for each task and each condition. Respiration data was down-sampled from 256 Hz to 120 Hz using MATLAB (The MathWorks Inc.) to match the rate of the motion capture data. All data was then imported in custom-made software for visualization and further processing.

To extract spontaneous synchrony in the first part of the experiment, we detected the footsteps from the four markers placed on each foot, taking as criterion that the foot was on the floor when its instantaneous speed was null. We then extracted a *footstep synchrony index* from the phase difference between footsteps. The formula used was the following:

$$FSI=\;Abs\left\{Min\left[Abs\left(\varphi^{P1}-\varphi^{P2}\right),Abs\left((\varphi^{P1}+1)-\varphi^{P2}\right),\;Abs\left((\varphi^{P1}-1)-\varphi^{P2}\right)\right]-0.25\right\}*4$$ (1)

**Fig 4:**
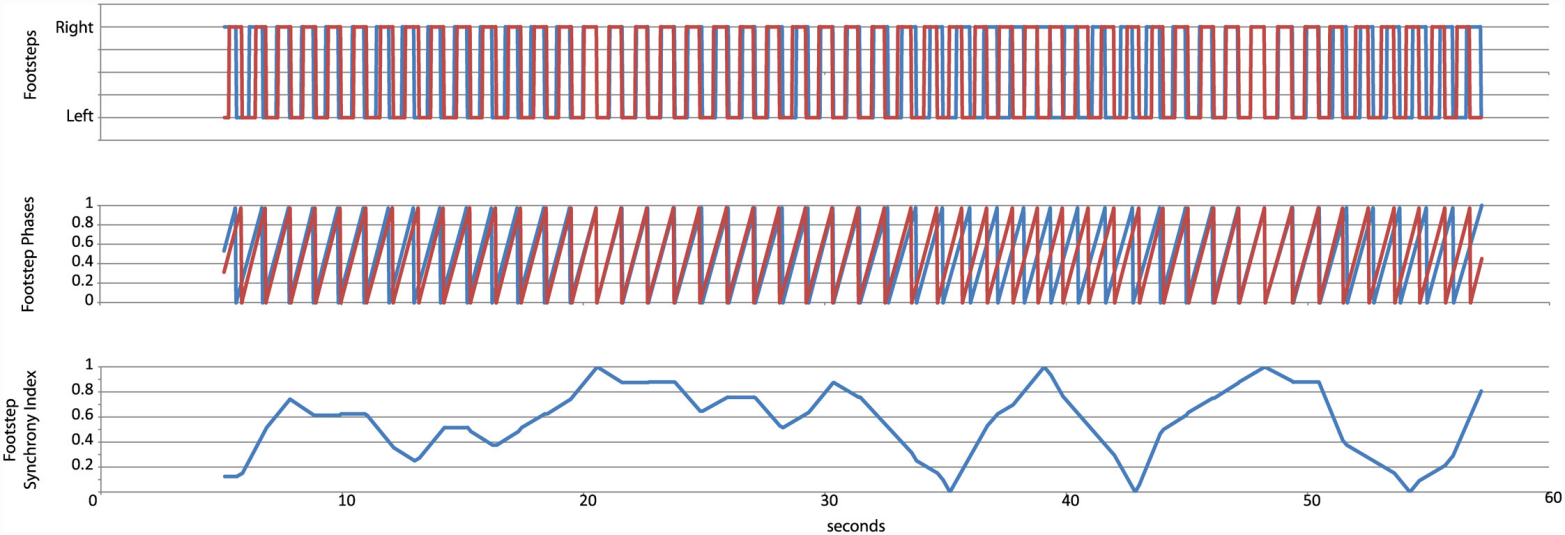
Extraction of the footstep synchrony index.

Where *φ*^P1^ is the phase for each participant between two footsteps, expressed between 0 and 1. As shown in Fig 4, the footstep synchrony index of 1 was obtained when both participants placed their foot on the floor simultaneously, either the same foot or the contrary one. A footstep difference index of 0 was obtained when the timing of the footsteps was maximally different (equivalent to 90° of phase difference). From this index, two measures were considered: the mean footstep synchrony index across a trial and the ratio of time on which the footstep synchrony index was over a certain threshold, which we experimentally selected at 0.7 (equivalent to 27° degrees of phase difference, quite smaller than 45°).

To extract respiration synchrony for each trial in the second part of the experiment, a *respiration synchrony index* was extracted using the same mathematical expression than for footsteps. The same two measures were also considered: the mean phase difference index across a trial and the ratio of time on which the respiration synchrony index was over 0.7.

To assess the behavioral synchronization of each couple during the different tasks, the hand positions of both participants were computed for each time instant. We used the two markers placed on each hand and averaged their positions to calculate the 3D hand coordinates (*x*, *y*, *z*). This position was then projected on the medial plane separating the two participants standing face-to-face, resulting in 2D coordinates (*x*, *y*). From the left and right hand positions of both participants on the medial plane, we calculated two movement descriptors, the *hands distance* (*h_dist*) and the *hands speed difference* (*h_speed_diff*), adapted from [12]. The first intended to capture the difference in the hands position at a given moment, and the second intended to capture the difference in hand velocity at a given moment. They can be formally expressed as follows:

$$h\_dist=\;\frac{\left||h_{left}^{P1}-h_{right}^{P2}||+||h_{right}^{P1}-h_{left}^{P2}|\right|}{2}$$ (2)

$$h\_speed\_diff=\frac{1}{2}\frac{\left||s_{left}^{P1}-s_{right}^{P2}|\right|}{\left||s_{left}^{P1}+s_{right}^{P2}|\right|}+\frac{1}{2}\frac{\left||s_{right}^{P1}-s_{left}^{P2}|\right|}{\left||s_{right}^{P1}+s_{left}^{P2}|\right|}$$ (3)

Were $h_{left/right}^{Pi}$ is the 2D projection of the *left* or *right* hand position of participant *i* on the medial plane and $s_{left/right}^{Pi}$ is the speed vector of the *left* or *right* hand of participant *i* in the medial plane. The average value was then computed for all measures at increments of 1 second, the temporal precision of the subjective reports.

### Statistical analysis

The differences between the *blind* and *joint* conditions were analyzed with repeated measures ANOVA using SPSS software (IBM SPSS Statistics; version 21.0)

In the joint experiment, to analyze the different factors affecting the subjective sensation of synchrony we first considered subjective factors. These were factors which were different between individuals, or between couples, but which did not change across trials. The factors considered were: positive PANAS score, negative PANAS score, empathy score, and the measures of spontaneous behavioral synchrony obtained in the first part of the experiment (which was common for both members of each couple).

To pre-select the subjective factors that could significantly contribute to the sensation of synchrony across a whole trial, we considered, for each 1 minute trial, the *total amount of time* in which participants felt in synchrony. To have a first assessment of whether the subjective sensation of synchrony was sensitive to each of these scores, the subjective reports of synchrony were correlated with these values. The results showing a significant correlation were then used as predictor candidates within the statistical model built to explain the instantaneous feeling of synchrony.

For the data related to the subjective sensation of synchrony, we built a generalized linear model [22] to predict the instantaneous feeling of synchrony, i. e., whether at a given instant the participant was having or not the subjective sensation of synchrony, and assumed it was the result of a Logit distribution. The relation between dyads and individual subjects was modeled as a multilevel model (see also [23]).

The possible predictors considered were the *time* since the beginning of the task, the *task* being performed, the subjective factors, as well as performance factors, such as *hand distance* and *hand speed difference*. We then built a simplified model considering only the criteria significantly (p<0.05) predicting the subjective feeling of synchrony (Table 1), and compared them with a likelihood ratio test.

**Table 1:** The main factors contributing to the subjective sensation of synchrony. The coefficient is the main number reported, the standard error is in parenthesis. The first model includes all the predictor candidates, and the second removes the non-significant ones.

	Model 1	Model 2
Fixed effects		
Intercept	-6.881** (2.348)	-3.344** (0.376)
Hand distance	-0.004* (0.001)	-0.004** (0.001)
Hands speed difference	-0.889** (0.292)	-0.866**(0.292)
Empathy score	0.077 (0.040)	
PANAS negative score	-0.117 (0.088)	
Gender	0.233 (0.392)	
0 ≤ Time <20	3.226** (0.228)	3.211**(0.227)
20 ≤ Time < 30	4.806** (0.233)	4.791**(0.232)
30 ≤ Time < 40	5.660** (0.239)	5.645**(0.237)
40 ≤ Time < 50	5.535** (0.237)	5.520**(0.236)
50 ≤ Time < 60	5.506** (0.238)	5.491**(0.237)
Task 2	-0.146 (0.115)	-0.146(0.115)
Task 3	0.374** (0.116)	0.376**(0.116)
Task 4	0.376** (0.117)	0.378**(0.117)
Task 5	0.540** (0.116)	0.541**(0.116)
Task 6	0.508** (0.117)	0.509**(0.117)
Random effects		
Level 1 (residual)	0.051	0.534
Level 2 (residual)	0.714	0.553
AIC	5727.6	5726.1
BIC	5849.9	5827.9
Deviance	5691.6	5696.1
	χ^2^(3) = 4.47, p = 0.21	

*p < 0.05,

**p < 0.01

For the data related to the video test session, we built a first generalized linear mixed model [22, 24, 25] to evaluate how a viewer is able to determine the instant at which both members of a couple are in synchrony (question Q1 of the test).

To relate the video test reports with the subjective reports on synchrony feeling, we built a predictor variable named *trigger*. The reason to introduce such predictor variable was because the subjective reports on synchrony sometimes had short onsets. In addition, one of the conditions to validate a trial was that the feeling of synchrony lasted more than 10 seconds. Therefore, since both participants had given different responses to the moment where they perceived to be in synchrony, the predictor variable named *trigger* was calculated from performing a principal component analysis of four different inputs. For each participant in the couple, we considered the first time they reported the sensation during the task, and the time they reported the sensation that validated the trial (i.e, when this sensation occurred during a common phase of at least 10 seconds, which could be different from the first time the sensation occurred). Additional predictor variables considered were: the task (*task*), the fact that the respondent was present or not in the video (*presence*), the gender of the respondent (*gender*) and the level of empathy of the respondent (*empathy*). In addition, we built a simplified model in which we have only retained significant predictors. The full model and the simplified model were compared using a likelihood ratio test (Table 2).

**Table 2:** Modeling of responses to the question Q1. This table addresses the moment an observer estimated whether a couple is in synchrony.

	Model 1	Model 2
Fixed effects		
Intercept	17.247 (22.786)	17.018** (2.436)
Trigger	0.160** (0.052)	0.183** (0.052)
Task2	1.957 (1.548)	
Task3	-0.969 (1.540)	
Task4	-0.753 (1.513)	
Task5	-1.357 (1.543)	
Task6	-1.890 (1.511)	
Present	-1.638 (1.284)	
Gender (male)	-1.142 (2.327)	
Empathy	0.027 (0.371)	
Random effects		
Level 1 (residual)	117.95	
Level 2 (individual)	72.55	
AIC	4746.5	4740.0
BIC	4799.6	4757.7
Deviance	4722.5	4732.0
	χ^2^(8) = 9.511, p = 0.301	

*n* (Level 1) = 614. *n* (Level 2) = 16

**p < 0.01

To find out if participants recognized themselves (question Q2 of the test), we attempted to model their answers by using a logistic mixed effects model [26]. We built a first model that included explanatory variables: *presence*, *task*, *gender* and *empathy*. According to the same strategy as above, we also built a second simplified model that only included significant predictors. Both models were then compared using the likelihood ratio test (Table 3). To analyze Q3, we also built a logistic mixed effects model, where the criterion was the appropriateness of the answer to Q2, and the explanatory variable the degree of confidence reported in Q3.

**Table 3:** Modeling of responses to the question Q2. This table addresses whether participants recognized themselves.

	Model 1	Model 2
Fixed effects		
Intercept	-2.311 (1.579)	-1.319** (0.161)
Present	0.653** (0.244)	0.642** (0.243)
Task2	-0.034 (0.323)	
Task3	0.106 (0.323)	
Task4	0.073 (0.317)	
Task5	-0.341 (0.341)	
Task6	0.295 (0.314)	
Gender (male)	-0.168 (0.278)	
Empathy	0.017 (0.025)	
Random effects		
Level 2 (individual)	0.210	0.231
AIC	735.32	726.56
BIC	780.66	740.07
Deviance	715.62	720.56
	χ^2^(7) = 4.942, p = 0.667	

*n* (Level 1) = 668. *n* (Level 2) = 16

**p < 0.01

The statistical analyses were performed with the software package R (R Core Team, 2014), version 3.1.1 and the Stata software (Stata SE 13.1, Revision Oct 2014).

## Results

### Task performance

Regarding the first part of the experiment, the mean footstep synchrony index across all trials was 0.56 ± 0.11 (mean ± SD), and the ratio of time were the synchrony index was above 0.7 was 0.40 ± 0.17 (mean ± SD). This suggests that subjects had a tendency to spontaneously synchronize their behavior.

**Fig 5:**
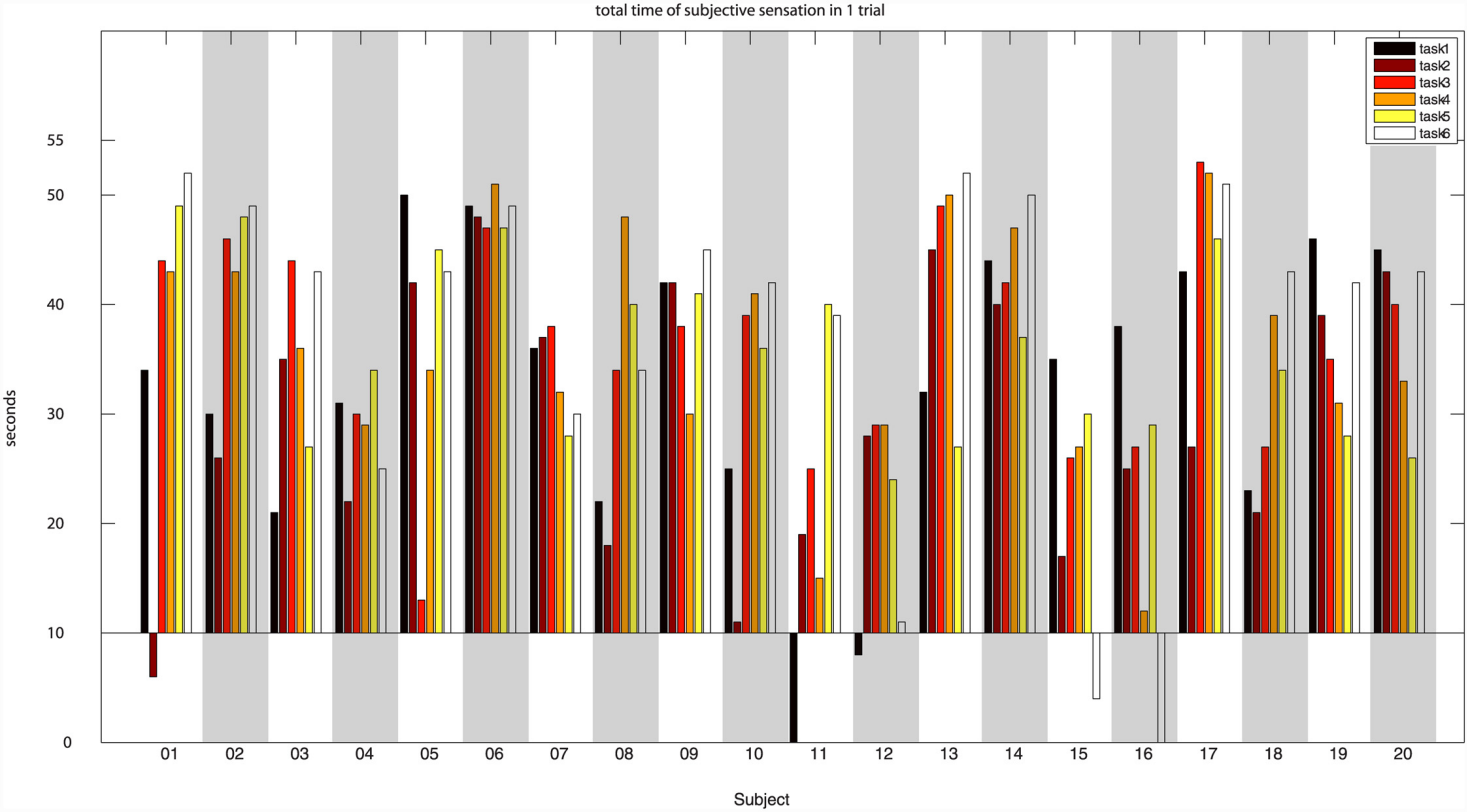
Total time of subjective synchrony per subject and trial.

Subjects were also able to perform the task and felt synchrony often largely over 10 seconds periods (see Fig 5). Tasks were mostly performed on the first trial (66,7%), and the mean number of trials was 1.7 ± 1.03. In few cases (1.7%), even after 4 repetitions, participants were not able to feel synchrony in a given task. In this case, the last trial was used. There were also few cases were participants felt synchrony for 10 seconds, but part of this period felt after the 1 minute considered as the main measure (see Fig 5).

### Joint versus Blind Task Performance

There were significant differences between the same tasks when done with or without visual cues. Indeed, comparing the *blind* vs. the *joint* conditions showed that the hands distance and the hands speed difference were considerably higher when participants performed the tasks during the *blind* condition compared to the *joint* condition (hands distance, *blind*: 279.14 ± 10.8 mm vs. *joint*: 104.3 ± 4.7 mm; hands speed difference, *blind*: 1.77 ± 0.15 m/s vs. *joint*: 0.28 ± 0.02 m/s). The difference between conditions was significant for both hands distance (F(1,9) = 276.7; p < 0.0001) and hands speed difference ((F(1,9) = 67.28; p < 0.0001) as shown by a repeated measures ANOVA.

For the respiration, comparing the *blind* and joint conditions showed no significant difference: *joint* (0.5± 0.019) and *blind* (0.5 ± 0.022) for mean phase differences (F(1,9) = 0.005; P = 0.94). There was also no difference between *joint* (0.3 ± 0.02) and blind (0.3 ± 0.03) conditions for the percent of phase synchrony over 0.7, as confirmed by a repeated measure ANOVA *(F(1*, *9) = 0*.*028; P = 0*.*871)*. Since respiration did not show any sensitivity to the fact that the task was done jointly or separately, we exclude respiration data from further analysis.

### Dynamics

Two subjective factors showed a significant correlation with the *total amount of time* in which participants felt in synchrony: the empathy score (r = 0.22, p = 0.015), and the negative PANAS score (r = -0.19, p = 0.036). On the contrary, the positive PANAS score, as well as the two measures of spontaneous synchrony extracted from the footstep synchrony index did not show a significant correlation. Within-dyad correlation of the total amount of time in which participants felt in synchrony was strong (r = 0.49, p<0.001). Empathy showed no significant sex difference (F(1,18) = 22.05; P = 0.47).

**Fig 6:**
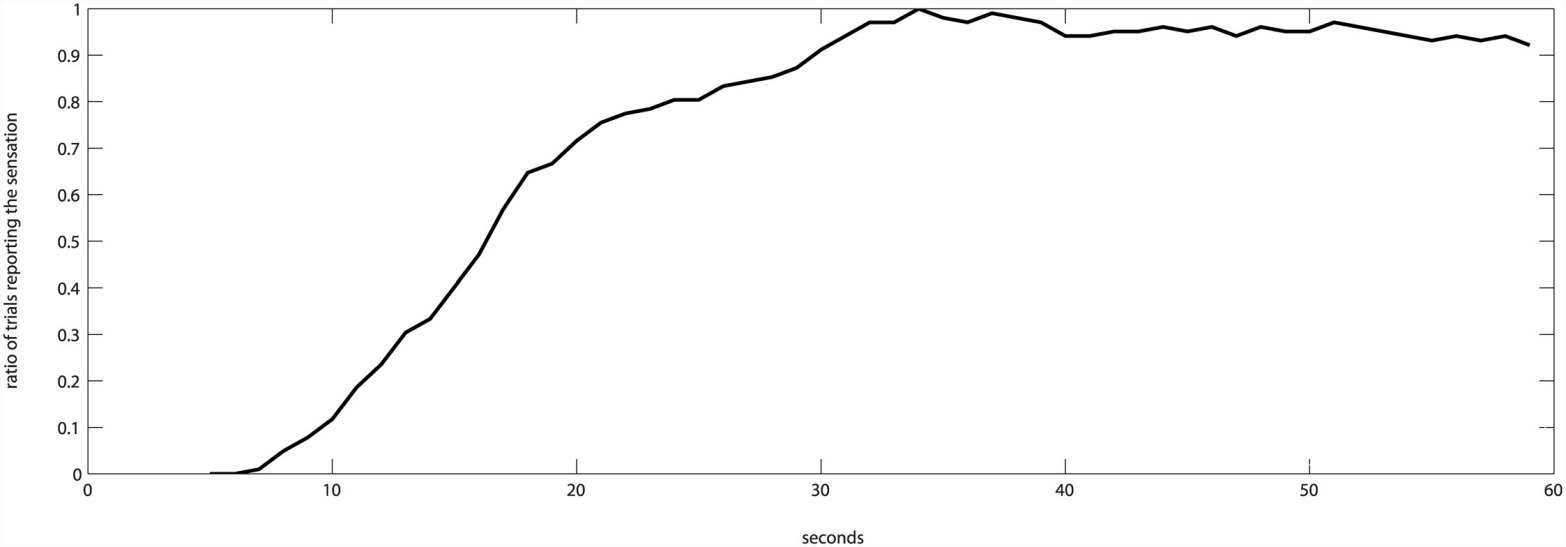
The ratio of subjective feeling of synchrony across trials shows a non-linear relation with time.

Since *time* showed a non-linear relation with the subjective sensation of synchrony (Fig 6), we modeled it as a set of categorical variables, for different segments of time (see Table 1). This method allows observing how the subjective sensation of synchrony evolves across time without imposing a shape to the function (see, for example, [27], when they propose a *growth model with an unspecified growth function*, *or* [28] when they address *non-linear curve fitting*). Even with this, it showed a very significant predictor of the subjective sensation of synchrony (Table 1).

*Time*, *task*, *hand distance* and *hand speed difference* significantly contribute to the feeling of synchrony. Despite it is not significant, in the extended model the empathy factor is near to significance (z = 1.93, p = 0.053). The simplified model, using only the significant factors, is not significantly better than the full model (χ^2^(3) = 4.47, p = 0.21).

### Video test session

Concerning the participants’ ability to identify the instant when the dyad was in synchrony, only the *trigger* variable corresponding to the moment when both partners were synchronized was significant among all potentially explanatory variables of the participants’ response to the question Q1 (Table 2). The simplified model using the *trigger* variable only fitted the data as well as the full model (χ^2^(8) = 9.511, p = 0.301). Neither the task nor being present in the video nor the gender nor the level of empathy improved the description of the answers to the question Q1.

Concerning the question Q2, the participants had difficulty in recognizing themselves. Of all the explanatory variables introduced in the first model, only the *presence* variable played a significant role (Table 3). The simplified model containing this variable only described the observed data as well as the full model (χ^2^(7) = 4.942, p = 0.667). The probability of responding ‘Yes, I am present in the video’ was influenced by the fact that the respondent was actually there or not. If the subject was present in the video, the probability that he reported ‘yes, I recognize myself’ was equal to 0.337 (95% CI [0.233, 0.453]), whereas it was equal to 0.211 (95% CI [0.157, 0.269]) if the subject was not present in the video. Moreover, it is important to note that each participant was present one time on seven among the videos presented.

Finally, responses to Q3 seemed to appropriately reflect the level of confidence that participants had in their responses. Indeed, their level of confidence in answering Q3 was significantly higher when they answered correctly to Q2 than when they did not (χ^2^(1) = 19.582, p < .001).

## Discussion

### Factors affecting the sensation of synchrony

The results of this experiment suggest that the subjective sensation of synchrony is affected by performance factors that can be extracted from the recorded movements.

The different factors affected the sensation in a direction that would seem intuitively obvious: the more similar the movements were, the sooner the sensation appeared. However, the relevance of such results should not be dismissed by the fact they seem intuitively obvious. Despite the *hands distance* is a movement descriptor that is only relevant for this concrete task, the *hands speed difference* is a movement descriptor that can be applied for quite general interactions. It could therefore be used to estimate the subjective sensation of synchrony in joint tasks not involving direct imitation. It should also be pointed out that the measures of spontaneous synchrony of footsteps in the first task did not correlate with the total time participants experimented synchrony in task 2. This suggests that subjective synchrony and spontaneous behavioral synchrony do not have a direct relation. This is consistent with [16], where two people spontaneously synchronizing their behavior in rocking chairs felt higher subjective synchrony when music was present, despite the behavioral synchrony was lower.

Since the negative PANAS and the empathy scores showed a clear correlation with the total time of synchrony sensation, it is possible that negative mood lowers the probability of the synchrony sensation, and that high empathy facilitates this sensation. This seems consistent with previous studies [29, 30], which relate the subjective feeling of synchrony with positive feelings, presumably caused by the participants’ assumption of having shared a common experience with each other. However, if it is the case the effect is smaller than the performance-related factors. Despite both showed a significant correlation with the total time of synchrony felt, they did not appear as significant factors in the overall statistical model. Empathy, in particular, was near to significance (z = 1.93, p = 0.053) and it is possible that it has a small but relevant effect. Further work to clarify the importance of these factors would ideally involve a simpler experimental setup allowing a more direct test of the role of empathy and negative affect.

On the other hand, factors commonly related with joint action tasks, such as spontaneous interpersonal synchronization of behavior or of respiration, do not seem useful to predict the extent at which people report subjective synchrony. This is particularly surprising in the case of respiration, since we could not even find significant differences between the blind and the joint conditions. One possible explanation is that the quite physical nature of the task did interfere with a mechanism inducing spontaneous synchronization of respiration.

However, we can also consider a different explanation. Previous work has reported spontaneous synchronization in collective groups [3], as well as in choir singing [13], and in a romantic relationship [31]. The study in [3] also shows that synchronization increased across sessions. It is therefore possible that physiological synchronization is not a short term phenomenon related with a joint task but rather a mechanism that is established in a longer term, as the result of bonding or familiarity. If this were to be the case, when couples in a relationship would perform the joint task they should have stronger levels of respiration synchronization.

During the video sessions, subjects were able to recognize the moment of synchrony as reported by the people doing the motor tasks. However, they were not very good in finding if they were or not in the video. This is in contrast with previous work showing people can recognize relatives and gender only from light point videos [32–34]. We attribute this difference to the nature of the task, requesting a specific movement, and not allowing for potentially characteristic features, like gait, or spontaneous expressions.

In addition, they were not better to identify the synchrony time if they were or not in the video. In other terms: an external observer seeing both participants’ movements on equal grounds, can estimate the joint performance of the couple, and therefore estimate the appearance of the sensation. However, to have the subjective feeling of synchrony with someone else, it seems necessary to move, and have the impression of performing in coordination with someone else. This is why the feeling seems to be affected by the task.

Previous studies showed that people performing together would spontaneously synchronize their behavior [16], peripheral physiology [13, 14] and even neurophysiologic activity [10], even if not asked explicitly to do so, or being aware that this synchrony is in place. One could therefore ask what would be the advantage of being aware of such a synchrony.

### How do we become aware of the sensation of synchrony?

The idea that awareness arises from synchronized brain activity between different cortical regions builds on the principle of perceptual binding [35–38]. To put in very schematic terms: synchronized brain activity is, at first, local. Progressively, different perceptive areas synchronize and generate a coherent multimodal percept. However, once this synchronization extends beyond perception areas, awareness arises. It has also been shown that synchronized brain activity appears when there is synchrony in joint tasks [10, 11]. It could therefore be speculated that since the joint behavior seems to imply joint neurophysiological activity, the synchronized neural activity in participants is responsible for this subjective feeling of synchrony. However, previous studies [1, 16, 39] showed that people tended to synchronize their activity even when not being requested to do so or being aware of it.

We therefore believe that behavioral or interbrain synchronization alone cannot account for the subjective sensation of synchrony. Alternatively, we believe it is possible to introduce a mechanistic explanation of the subjective feeling of synchrony if, instead, we turn to the theory of Graziano [40], who postulates that awareness is the result of a cognitive process focused on monitoring attention. Monitoring attention in others is an important part of our social skills and, according to this theory, when this same mechanism is applied to ourselves it generates the feeling of consciousness, the feeling of “being aware of”.

If we assume that attention-monitoring mechanisms are necessary for subjective awareness, then the subjective feeling of synchrony can be identified with a set of factors. It would occur when our attention-monitoring mechanism reports that both our attention and the other’s attention is focused on a concrete shared task, and that this task involves activity performed in a reasonable level of behavioral synchrony. This could readily generalize to everyday situations where, despite the absence of an explicit task that requires focused attention, gaze, facial expressions and social cues allow inferring much more precisely the attentional state of other people in a social context. We therefore believe that Graziano [40]’s theory offers a good interpretation of the results and helps explaining the existence of such a subjective sensation of synchrony.

In future work, we will explore the sensitivity of this behavioral model to population variation (married couples, musicians or actors, etc.). In addition, we will also explore whether we can elucidate the brain mechanisms supporting this sensation.

## Supporting Information

S1 Video Demonstration video of the main experimental task. (MP4)

S1 Dataset Dataset used for statistical models. (XLSX)
